# Universal Health Coverage in Bangladesh: Activities, Challenges, and Suggestions

**DOI:** 10.1155/2019/4954095

**Published:** 2019-03-03

**Authors:** Taufique Joarder, Tahrim Z. Chaudhury, Ishtiaq Mannan

**Affiliations:** 1 Bangladesh Office, FHI 360, Dhaka 1213, Bangladesh; 2 Bangladesh Country Office, Save the Children, Dhaka 1212, Bangladesh

## Abstract

Catastrophic health expenditure forces 5.7 million Bangladeshis into poverty. Inequity is present in most of health indicators across social, economic, and demographic parameters. This study explores the existing health policy environment and current activities to further the progress towards Universal Health Coverage (UHC) and the challenges faced in these endeavors. This qualitative study involved document reviews (n=22) and key informant interviews (KII, n=15). Thematic analysis of texts (themes: activities around UHC, implementation barriers, suggestions) was done using the manual coding technique. We found that Bangladesh has a comprehensive set of policies for UHC, e.g., a health-financing strategy and staged recommendations for pooling of funds to create a national health insurance scheme and expand financial protection for health. Progress has been made in a number of areas including the roll out of the essential package of health services for all, expansion of access to primary health care services (support by donors), and the piloting of health insurance which has been piloted in three sub districts. Political commitment for these areas is strong. However, there are barriers pertaining to the larger policy level which includes a rigid public financing structure dating from the colonial era. While others pertain to the health sector’s implementation shortfalls including issues of human resources, political interference, monitoring, and supervision, most key informants discussed demand-side barriers too, such as sociocultural disinclination, historical mistrust, and lack of empowerment. To overcome these, several policies have been recommended, e.g., redesigning the public finance structure, improving governance and regulatory mechanism, specifying code of conduct for service providers, introducing health-financing reform, and collaborating with different sectors. To address the implementation barriers, recommendations include improving service quality, strengthening overall health systems, improving health service management, and improving monitoring and supervision. Addressing demand-side barriers, such as patient education and community empowerment, is also needed. Research and advocacy are required to address crosscutting barriers such as the lack of common understanding of UHC.

## 1 Introduction

Universal Health Coverage (UHC) implies that all people have access to quality health services they need, without financial hardship [1]UHC received a fresh momentum with the adoption of Sustainable Development Goals (SDG), the eighth target of the third goal of which states, “Achieve UHC, including financial risk protection, access to quality essential healthcare services and access to safe, effective, quality and affordable essential medicines and vaccines for all” [2].

The health system of Bangladesh is experiencing a double burden of diseases, low service coverage, and a lack of effective financial risk protection mechanism. Bangladesh has a pluralistic healthcare system, which is highly unregulated and consists mainly of four key actors: government, for-profit private sector, not-for-profit private sector (mainly the nongovernmental organizations [NGOs]), and the international development organizations [3]. Public healthcare is steered by the Ministry of Health and Family Welfare, through its different Directorate Generals: Health Services, Family Planning, Drug Administration, Nursing and Midwifery, Health Economics Unit, etc. [4]. Private healthcare encompasses for-profit private, not-for-profit private (mainly the NGOs), and informal providers (village doctors and other vast array of different unqualified providers). The public healthcare services are organized along four levels: community level healthcare (provided by the domiciliary health providers and community clinics), primary level healthcare (provided in Rural Health Centers, Union Subcenters, Union Family Welfare Centers, and Upazila Health Complexes), secondary level healthcare (provided in District Hospitals, General Hospitals, Chest Disease Clinics, Tuberculosis Clinics, and Leprosy Hospitals), and tertiary level healthcare (provided in Post Graduate Medical Institutes, Specialized Healthcare Centers, Medical College Hospitals, and Infectious Disease Hospitals). The private sector also has health facilities ranging from individual doctors’ offices to high-end tertiary level international standard hospitals [4]. Public healthcare is highly subsidized by the government, with nominal payments required from patients, especially for the outpatient care. Health insurance, both national and private, is practically nonexistent. Health financing is underfunded; only 2.64 percent of gross domestic product (GDP) is spent on health, which is the lowest in the south Asia region [5]. Health financial coverage is so sparse that nine percent households face catastrophic health payment, 5.6 percent face impoverishment, and seven percent face distress financing (borrowing or selling household assets to finance healthcare costs) [6].

Equity in health is one of the central pillars for promoting UHC 7According to the International Society for Equity in Health, “Equity is the absence of systematic and potentially remediable differences in one or more aspects of health across populations or population groups defined socially, economically, demographically, or geographically” [8]. Unfortunately, out of pocket (OOP) contributions to health expenditure, one of the most inequitable sources of healthcare financing, in Bangladesh, are among the highest in the world with 67% 9Quality of care, another important dimension of UHC, is highly questionable in the public sector [10]. This encourages people to resort to private sector healthcare, which is more expensive. Health expenditure in private health facilities is almost exclusively from OOP payments (93%) 11

The review of Bangladesh’s Demographic and Health Survey 2014 reveals inequity in most of the health indicators in terms of economic status, level of education, gender, location (urban vs. rural), and geography (divisions) [12]. Among fertility and family planning indicators, for example, marital age of first marriage is only 15.3 years in the lowest income quintile versus 17.6 years in the highest (national average 16.1 years). Mean ideal number of children is 2.4 among women with no education versus 2.0 among those with secondary or higher level of education (national average 2.2). Contraceptive prevalence rate (any method) is only 47.8% in Sylhet Division versus 69.8% in Rangpur (national average 62.4%). Percentage of unmet needs for family planning is 17.7 in the Sylhet Division versus 6.7 in Rangpur (national average 12.0).

Similar trends of inequity are observed in maternal and child health and nutrition indicators as well. For example, infant mortality rate is 35 per 1000 live births among the people of lowest income quintile, compared to only 14 among the highest income group. Antenatal care (ANC) coverage rate is highly inequitable in terms of all types of stratifications; for example, there are 14.7, 25.7, 37.6, and 37.8 percentage point differences between urban vs. rural, Khulna division vs. Sylhet division, completing secondary or higher education vs. no education, and highest vs. lowest income quintiles, respectively 12

The Bangladeshi Constitution commits to address inequalities in access to health in rural areas, and the country joined the global community in committing to achieve UHC by 2030 under the SDGs 13 However, there is no single route or ‘magic bullet’ to achieve UHC. While each country must decide its own path towards UHC based on the individual country contexts, all should draw on existing evidence and shared experience. Currently available evidences from Bangladesh on UHC mostly include quantitative household surveys on out of pocket expenditure [14, 15], financial risk protection 6 15–17] and equity analysis [6, 16–18]. The policy environment around UHC issues has been analyzed by very few studies, which includes an assessment of a set of proposed indicators related to UHC [19, 20], and generic policy papers without description of methodology [21, 22]. None of these papers systematically analyzed the UHC policy using qualitative research methods, involving the stakeholders. Therefore, in this study, the aim was to understand the existing health policy environment and current activities to further the progress towards UHC and the barriers or challenges faced in these endeavors. Suggestions by different stakeholders were also explored for an in-depth understanding of UHC.

## 2 Materials and Methods

This qualitative study, conducted between May and June 2017, involved document reviews and key informant interviews (KII). In order to understand the existing policy environment around UHC, we adopted the UHC Cube framework for data generation and analysis [1]. We explored the activities of different stakeholders in light of population coverage, access to services and financial protection dimensions. Then, we explored the policy, intervention-, and demand-side barriers pertaining to the three UHC dimensions. Inductively, we explored some barriers, which cut across all three levels. Finally, we explored the stakeholders’ perspective on how they want to address the barriers that they had identified.

Document reviews included published reports, guidelines, strategic documents, and policy documents. An initial list was prepared first, which was later supplemented by the information and suggestions from the informants of the KIIs. Reference tracking of published articles, consulted during the literature review, also contributed to the list.

KIIs were conducted using semistructured guidelines, supplemented by qualitative probing techniques. A tentative list of potential key informants was developed with consultation among the research team (a health systems researcher with experience in UHC related academic activities in Bangladesh, personnel from an international Nongovernmental Organization NGO involved in UHC advocacy, and a senior leader of an international NGO involved in implementing public health programs and health systems strengthening). The list was supplemented by snowball recruitment techniques as the interviews progressed.

Key informants were sampled purposively, aiming for maximum variation [23] in terms of their sectoral alignment. In total, 15 respondents were interviewed from different sectors, broadly categorized as follows:

1. Public sector (central level): 1
2. Public sector (district level): 5
3. Multilateral organization/donor: 2
4. NGO/implementation organization: 4
5. Academia/research: 2
6. Civil society (health journalist): 1

Interviews were conducted in the interviewees’ office, by two public health experts (one male and one female). The key informants were assured of strict anonymity regarding the content of their interviews. All the interviews were digitally recorded; however, they were provided the option of speaking ‘off the record’, should this be preferred. Manual note taking was also employed for all interviews in order to prevent the risk of data loss due to technical issues.

Although a key informant guideline was used for data generation, adequate probing was used to clarify the views expressed by the respondents. Follow-up questions were asked to ensure maximum and in-depth information. Each interview lasted between 25 and 45 minutes. The interview tool included questions on activities carried out by the respondent’s institution in light of UHC across the three dimensions of UHC: population coverage, access to services, financial protection [1], perceived barriers towards carrying out those activities, and suggestions to overcome those barriers.

Verbatim transcriptions were done by professional transcribers once the interviews had been finished. The transcripts were subsequently read carefully and matched with the records, to determine missing information. The thematic analysis of texts was done using manual coding. Texts were organized across three main themes: (1) activities around UHC, (2) barriers to implement UHC, and (3) suggestions to progress towards UHC. Appropriate quotations were extracted to substantiate the thematic analysis. Member checking was done through a seminar presentation with the key informants to ensure that their views are correctly and adequately reflected. To increase validity, the first and the second authors independently coded the dataset. The third author was involved where a third opinion was warranted to reach consensus or resolve controversial issues.

## 3 Results

### 3.1 Policy Scan

The document review, aiming at understanding the existing policy environment, included 22 documents and a renowned journal’s special series on UHC issues in Bangladesh. Among the 22 documents, majority (n = 16) were published by different entities of the Government of Bangladesh (GoB), especially Ministry of Health and Family Welfare (MoHFW). The remaining were published by multi-lateral international organizations, civil society consortiums, and private academic and research organizations (complete list and findings in supplementary information file 1).

In order to address inequities and foster UHC, the GoB has taken several policy initiatives. Besides, various multilateral organizations, civil society consortiums, and academic and research organizations based in Bangladesh developed documents with policy directives for UHC in Bangladesh. We classified the GoB policy documents as follows: (1) overarching documents, not specific to the health sector; (2) overarching documents specific to the health sector, but not specific to health financing; (3) documents specifically related to health financing; and (4) documents not directly related to, but with implications for UHC (supplementary information file 1).

The most important policy document, specifically focusing on UHC in Bangladesh, is the ‘Health Care Financing Strategy 2012-2032: Expanding Social Protection for Health towards Universal Coverage’, published by the Health Economics Unit (HEU) of MoHFW [24]. Aligned with other important policy documents (e.g., National Health Policy 2011 [25], Health Population and Nutrition Sector Development Program (HPNSDP) 2011-2016 26etc.), this strategy document acknowledged the importance of bringing more funds to the health sector and pooling the resources effectively. It summarized challenges of health financing in Bangladesh as (1) inadequate health financing; (2) inequity in health financing and utilization; and (3) inefficient use of existing resources. Designed to address the health-financing issues for the next 20 years, this document also proposed ways to combine funds from tax-based budgets with proposed social health protection schemes (including for the poor and the formal sector), existing community based and other prepayment schemes and donor funding to ensure financial protection against health expenditures for all segments of the population, starting with the poorest. It recognized the importance of and proposed collaboration with the for-profit and not-for-profit private sector, development partners, and the community people, to resolve the health-financing challenges. It proposed a gradual process to achieve universal coverage, starting from the poor and the formal sector (public, for-profit private, and not-for-profit private), progressively to remaining segments of the population by 2032.

Apart from this crucial document, a few other important policy documents provided important policy directions for UHC in Bangladesh. For example, the ‘Seventh Five-Year Plan Fiscal Year 2016-2020: Accelerating Growth, Empowering Citizens’ 26 expressed commitment to ensure that poor and marginalized people are able to access and utilize health services. Acknowledging the existing deficiency in per capita health expenditure, share of the national budget for health, quality of care and high OOP, it proposed a health-financing reform to address these issues. In light of these proposals, the ‘National Social Security Strategy of Bangladesh’ [27] suggested some specific reforms and action plans and listed relevant ministries to collaborate with. It expressed the commitment of the GoB to introduce a national health insurance scheme. These reforms require budgetary allocation, the insufficiency of which has been recognized by the ‘National Health Policy’. It not only recommended increasing the allocation but also proposed ensuring equitable care for the disadvantaged, poor, marginalized, elderly, and the disabled population. In alignment with National Health Policy’s recommendations, the ‘Health Population and Nutrition Sector Strategic Plan 2011 – 2016’ [27] dedicated a chapter on ‘health sector financing’, where it proposed a health-financing framework, advocated demand-side financing, and proposed a resource allocation formula. The ‘Health Nutrition and Population Strategic Investment Plan 2016-2021’ [27] identified 10 driving forces, the final one of which suggested greater investment in health, ensuring a focus on managing demand, increasing efficiency, and developing the evidence base for future health funding. It also identified Essential Service Package (ESP) as the first milestone on the road to UHC. It proposed three guiding principles for attaining UHC: quality, equity, and efficiency across health services. Apart from these government documents, many nongovernment ones, such as ‘Bangladesh Health Watch Report 2011: Moving Towards Universal Health Coverage’ [28], and ‘The Path to Universal Health Care in Bangladesh: Bridging the Gap of Human Resources for Health’ [29], advocated for creating greater demand for UHC and quality primary health care (PHC) among the community through support from the civil society.

### 3.2 Current Activities towards UHC in Bangladesh

The information presented in this section has been obtained through both document reviews and the key informant interviews.

*Public Sector.* ESP has been identified as the basis for UHC activities in public sector of Bangladesh. ESP is currently in the process of implementation, even at the lowest unit of health service delivery facility, the Community Clinic (CC) level. A pilot health-financing scheme, Shasthyo Suroksha Karmasuchi (SSK), has been introduced by HEU in three upazilas (Kalihati, Ghatail, and Madhupur) of Tangail District [30]. Initially, the below-poverty population has been included in the scheme (includes treatment for 50+ disease conditions) with the government paying for their premium; the above-poverty population is also intended for gradual inclusion in the scheme.

At the district or implementation level, information and communication technologies (ICT) are used extensively to improve population coverage. Services are delivered through health centers as well as through household visits. Social and behavioral change communications (SBCC) and Expanded Program on Immunization (EPI) activities are carried out as preventive measures. Curative programs include Integrated Management of Childhood Illnesses (IMCI), maternal and neonatal health activities, demand-side financing programs (DSF) with vouchers, Emergency Obstetric Care (EOC), and indoor and outdoor services, in most areas, if not all. The family planning directorate of the MoHFW is also engaged in sexual and reproductive health care, in addition to their role in family planning. The government has started shifting focus from just quantity to the quality of services as well. Medicines are given free of cost from health centers, which reduces the financial burden of the patients to some extent.

*NGO Sector.* NGOs are mainly engaged in increasing service coverage and that with service quality. In terms of service coverage, their emphasis is on newborn health, maternal health, nutrition, health system strengthening, etc. at the PHC level. In terms of population coverage, their main focus is towards the hard-to-reach areas and the population therein. They are focusing on community engagement and SBCC activities, which may go a long way to demand generation among the population for UHC and also decrease the financial burden for curative caer. A respondent from an international NGO clarified this concept:

“If we strengthen the preventive care, if we strengthen the SBCC components, that actually is the best way to bring down the cost of treatment in future.”

They also advocate with the government for modifying policies, many of which directly or indirectly contribute to the UHC journey.

*Multilaterals and Donors.* Multilateral organizations, such as World Health Organization (WHO), are more into generating a common understanding on UHC among the stake-holders. They are also providing technical support to the government in implementing UHC. Generating information and strengthening the health system are their larger approach to contribute to UHC activities. Highlighting importance of multisectoral action for UHC, a representative of a multilateral organization remarked,

“It is a big area where our organization wants to work and make changes. We try to engage all the concerned ministries, often through dialogues.”

Donors supported the HEU in developing the Health Care Financing Strategy 2012-2032 and also its implementation [24]. Raising awareness and a common understanding on UHC has also been a main focus.

*Academia and Research.* Academia and research organizations’ role is to familiarize the concept of UHC to the relevant stakeholders. A professor of a school of public health remarked:

“We are trying to bring them (stakeholders) to the consensus, so that they are clear about what this (UHC) is, why is it necessary, what to do in order to achieve it, and how they all can contribute to this cause.”

They are organizing short courses to develop capacity, conduct research work on UHC related issues, and do policy advocacy through roundtable discussions, TV talk shows, etc. Research organizations are also involved in planning, monitoring, and evaluating GoB health programs relevant to UHC.

*Media.* The media is also involved, as expected, in awareness building about UHC, especially among the common mass. Media has been involved since the beginning of the UHC agenda in Bangladesh, starting from a grant from Rockefeller Foundation made to the quasigovernmental autonomous organization, Press Institute of Bangladesh (PIB). Journalists received training on UHC, are writing extensively on different aspects of UHC, and visited the SSK project. Some journalists even went to other countries (e.g., Thailand, Nepal, Bhutan, Philippines) on an exposure tour. A senior health journalist said:

“These exposure tours helped me develop an idea about what other countries are doing in terms of UHC. All I have been writing in newspapers, and what I am telling you now, are in light of these visits.”

PIB is regularly organizing training and orientation sessions for journalists, and TV talk shows on different aspects of the UHC agenda [31].

### 3.3 Barriers towards Achieving UHC in Bangladesh

**Figure 1:**
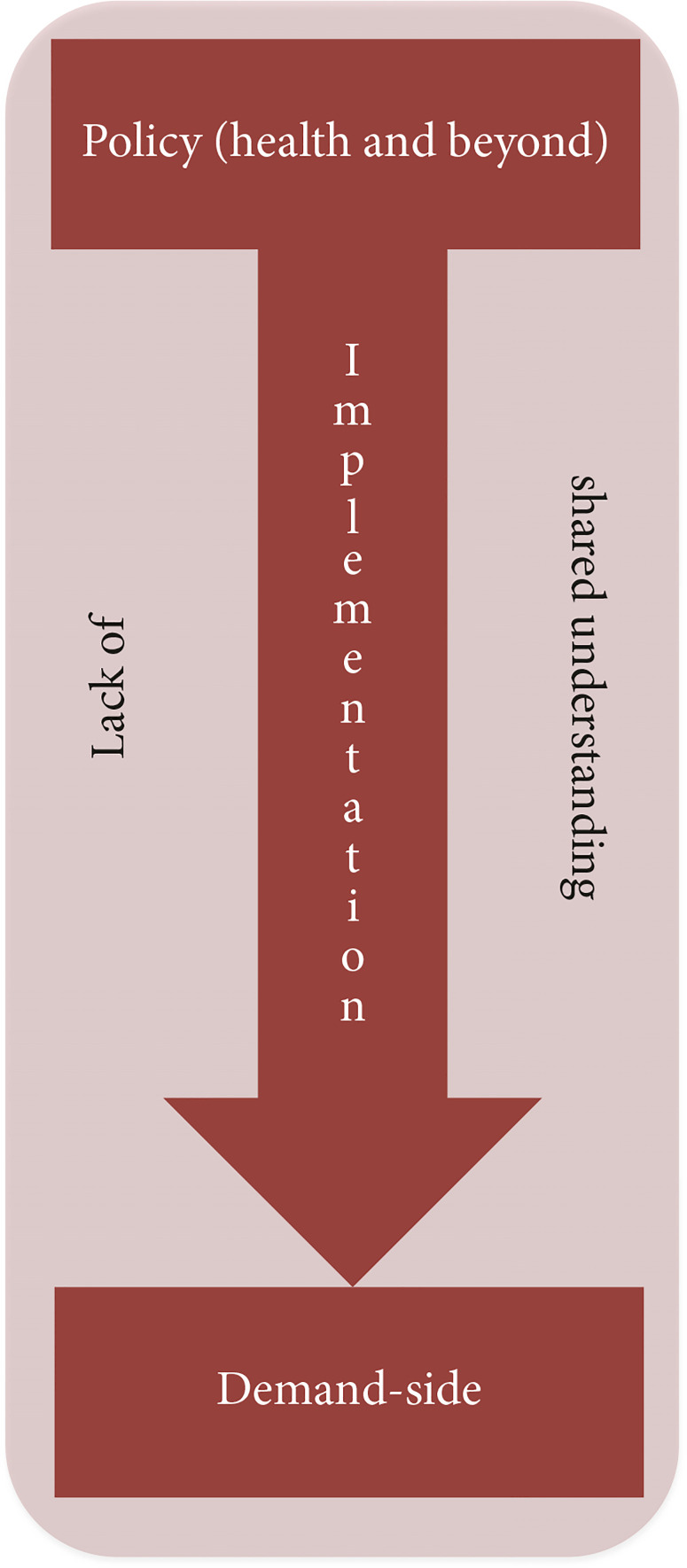
Barriers of implementing UHC in Bangladesh.

The information presented in this section has been obtained through key informant interviews. The barriers to progress towards UHC can be felt at different levels. The barriers have been categorized across three levels, which are again crosscut by one important barrier, the lack of a shared understanding on UHC. The three levels are (1) larger policy-level barriers, often beyond the jurisdiction of health sector alone, (2) implementation barriers in health sector, and (3) demand-side barriers (Figure 1).

*Larger Policy-Level Barriers (Health Sector and Beyond).* Public financial management has been designed such that only health sector finance is very difficult to alter separately. Ministry of Finance needs to change all its mechanisms and rules of procedures for all other ministries, if it wants to do something for one particular ministry. Bangladesh has traditionally been practicing supply-side budgeting, whose changing is complicated, has crosscutting ramifications, and, therefore, demands much broader or revolutionary commitment for whole system change. An expert from an international NGO on health systems strengthening said

“This is the legacy of British colonial bureaucracy, which no one dares changing, despite how much they want.”

In tandem with the increase in economic activities in people’s lives, the size of the economy is increasing. Since the purchasing power of people is increasing, their health seeking behavior is changing consequently, culminating in higher healthcare cost. A government high official said

“People nowadays go quickly to the doctor, demands quick diagnostic tests, want to get cured soon. They are much more aware. Some of this is informed awareness, which is good. However, there are some ill-informed care-seeking, which are increasing the cost of care unduly.”

As a result of all of these, the overall healthcare expenditure is increasing, which requires more funding to address. Eventually, implementation of UHC is becoming progressively expensive. This is happening in the context of a country that chronically allocates least share of its national budget for health. On top of all these, health insurance, if we consider as a means to UHC, itself is resource intensive.

The regulatory mechanism is not adequately functioning to regulate the private sector. There is currently no such structure for functional mediatory mechanism, to resolve or mediate the complaints of the service seekers. Unqualified providers often continue harmful medical practices, capitalizing loopholes in the regulatory framework and its implementation.

In addition, there is deficiency in health systems governance and stewardship. Accountability and transparency are difficult to ensure in public sector, especially in the presence of a highly centralized system. One key informant from a multilateral organization said,

“With the very centralized system it is difficult for the managers to be able to act on the information or data they have. So, they don’t always make decisions for their community, for their catchment area– based on the local data.”

*Implementation Barriers in Health Sector.* Poor human resource management, including shortages, deficient training, low motivation, retention issues, skill-mix imbalance, and quality service provision is staggering. Recruitment mechanisms by Bangladesh Civil Service (BCS) are also criticized for taking too long to deploy physicians into vacant posts in time. Political interference is often adding insult to injury, as recruitment, retention, and disciplinary measures become difficult for managers to exercise. One local level government health official said

“There are some bad areas near Hobiganj, where I cannot retain any physician. As soon as I deploy someone, phones keep coming from the honorable minister, secretary, political leaders, Awami League (ruling party) secretary, petit leaders, so and-so, requesting not to keep his doctor there.”

The same bureaucrat also reflected on difficulties of disciplinary actions against his employees due to political interference:

“Yesterday, going to an Upazila Health Complex I discovered, the physician had not come to his duty on time. I asked him to show the causes of his absence. Since evening I had received at least 10 phone calls, vouching for him that he had genuine reasons to remain absent that day. If I tend to take disciplinary actions, they would say, ‘why are you making too much of it?”

Deficient monitoring mechanisms often exacerbate the shortage of human resources, as the existing service providers cannot be ensured to stay in their posted positions.

There is no agreed-upon protocol for treatment, referral, follow-up, and even general service management. As a result, uniform care with sufficient quality is difficult to provide. Lack of proper training of service providers results in lack of quality of care and responsiveness towards the service seekers. Since the service providers are trained in a certain way, convincing them to do it differently overnight is challenging. Implementers of SSK in three upazilas faced this problem, as reported by a key informant from the central level of the government:

“They (service providers in SSK facilities) are trained in a certain way. When we are giving direction to do something differently for the sake of the project, they are saying, ‘why should I do it like this?’ Now, no one is willing to go to those centers, despite those being close to Dhaka. Someone even told me, if he has to stay there, he would rather leave the job.”

Identifying and reaching the hard-to-reach areas and population is another challenge to achieve UHC. Bangladesh has several difficult geographical regions, e.g., *char* or newly emerged land-strip in the bank of rivers, coastal areas, hill tracts, tea garden, etc. There are minority population groups, in terms of religion, ethnicity, etc. Also, the socially excluded and economically marginalized population needs special attention. Due to persistent lack of flexible budgeting and local level planning, these population groups never get due attention. One key informant from an international NGO said

“Government’s system is that; they always do flat budgeting. They allocate the same budget for hilly tea gardens of Sylhet, as they do for flat areas of Rangpur.”

Apart from the above-mentioned barriers, some key informants pointed to the donor dependency and consequent compromise in autonomy of priority setting, lack of sufficient infrastructure, management shortfall, elusive collaboration and coordination mechanism, to name a few.

*Demand-Side Barriers.* There is a pervasive sociocultural barrier against insurance. Several key informants mentioned there is overall deficiency of trust in the society, due to historical and sociocultural reasons. According to one key informant from the government sector:

“Let alone trusting health insurance agency, we don’t even trust our relatives, when it comes to financial transactions.”

Due to the pervasive lack of societal trust, coupled with lack of historical precedence to insurance mechanisms, it is difficult to convince people to give their money to a pooled fund.

Another historically established perception among the people is that the government is solely responsible for health and that must be free of cost. People are not receptive to the idea of paying for government healthcare, be it in the form of prepayment or otherwise.

Lack of information on the available services is another demand-side barrier to UHC. Communities also lack awareness regarding their own entitlements. They are not empowered enough to hold the decision-makers and services providers responsible for providing UHC.

*Crosscutting Barriers.* A lack of common understanding among different stakeholders, both supply and demandside, has been identified by several key informants as an overarching barrier to UHC. According to key informants, different people perceive UHC differently; for example, some think UHC is just about insurance (including commercial health insurance), some think this is just a variant of the PHC movement, while some others consider any activity pertaining to health as UHC. One key informant from an academic institution said

“If you ask a government officer, he would say, ‘we already have UHC’; healthcare has been free in Upazila Health Complexes since ages. We provide all types of health service; we don’t need a new UHC.”

Key informants suggested that if stakeholders lack a shared understanding and are not sufficiently motivated as a result, it would be difficult to take the UHC movement forward.

### 3.4 Suggestions from the Stakeholders to Overcome the Barriers

The recommendations are drawn from respondents and have been aligned with the barriers to UHC, as mentioned before.

Suggestions to Address Larger Policy-Level Barriers (Health Sector and Beyond)

*(1) Redesign the Public Financial Management.* Bangladesh must invest in addressing inequalities in access to health services and reducing reliance on OOP payments if it is to achieve UHC by 2030. Bangladesh’s Heath Nutrition and Population Strategic Investment Plan 2016-2021 recognizes the importance of investing in a strong foundation for UHC through its commitment to delivering primary healthcare under the ESP as the first milestone on the road to universal coverage. However, in order to accommodate the flexibility in financing options, required for UHC, redesigning the traditional public financial management is recommended. It should be rearranged in a way to accommodate demand-side financing, projects like SSK, local level planning, and local authority for spending.

*(2) Health Insurance and Health-Financing Reform.* The government should consider introducing a national single payer system and increase coverage gradually to different population segments; starting with the formal sector as they are more informed and more empowered to reclaim their right. Bangladesh needs its own model for health financing, which warrants further research and experiments. Creation of a purchasing body and separation of providers from purchaser authority is needed. Innovative financing mechanisms, such as bringing corporate social responsibility (CSR) money, *zakat* money, and sin tax money into UHC should be considered. A senior health journalist suggested

“There is no such corporate house which doesn’t do CSR. They can give this fund for UHC; only government needs to take the initiative. Zakat is also similar to CSR. If the government makes a law that people should contribute their zakat fund to UHC, that’s enough. It’s easy to convince people that ‘you are rich, but someone among your poor neighbors is deprived of treatment and dying The government makes arrangement with the tobacco industry that, their money is going to different sectors; this must be earmarked for health sector only.”

*(3) Improve Regulatory Framework and Mediatory Mechanisms*. It should be done with the aim of decreasing cost of medicines and healthcare. Policy makers need not only develop protocols, but also ensure compliance to these. Private sector should be regulated for better management, improved quality, and reduced cost. Regulation and its implementation should ensure that there is no overcharging, exploitation of any form, unnecessary procedures and tests, and irrational use of antibiotics. There should be an oversight board, or a private sector coordination board, and/or a functioning mediatory body. Reflecting on the high cost of medicine, a key informant from the government said

“They (pharmaceuticals) justify their high cost by saying, ‘Show me which country is giving medicine in lower price? Even you go to neighboring India, the price is higher there too’. But they never say that we can give medicines with much less price.”

*(4) Intersectoral Collaboration*. Civil society needs to be consulted for optimization of UHC endeavor. A key informant from the leadership of an international NGO said

“There should be a civil society and government collaboration, where we (civil society) will monitor our (national) progress, keep a watch, engage in dialogues, and raise our voice from time to time. This is needed so that the government, or whoever is working (on UHC), stays on right track.”

There needs to be an institutional body or a coordinating body, involving all relevant ministries or sectors and developing a common pathway towards UHC. It is important to include nonstate actors in the UHC movement, in order to get their data and insights. NGOs should come forward to support the government with technical expertise they have. A key informant from an international NGO said

“Role of organizations like ours is to raise the technical voice for UHC, and give courage to the government, stay with the government. We are not here to make them (government) look bad, rather we are trying to make their achievements more sustainable.”

Professional associations of service providers and the media should also be collaborated with. Collaborations should be addressed in the policy explicitly. There should be a clear guideline regarding intersectoral collaboration.

*(5) Political Commitment.* Political commitment and a better buy-in on UHC are indispensable. This may be achieved by going to the political parties before election and convincing them to include UHC in their manifesto.

Suggestions to Address Implementation Barriers in Health Sector

*(6) Health Systems Strengthening.* Comprehensive improvement in all health systems building blocks, such as financing, governance, and human resources, should be planned and operationalized. With an aim of overall health systems strengthening, PHC services should be prioritized, and duplication of services (between public and private sector, health and family planning, etc.) must be avoided.

*(7) Improve Health Service Management.* Health service management, including human resource management, inventory management, facility management, financial management, needs to be further improved. Vacant positions need to be filled.

*(8) Improve Monitoring and Supervision.* In order to improve supervision, the managers should get more support from the government; e.g., they should get vehicles and communication cost, etc.

*(9) Involve ICT.* Government of Bangladesh has placed importance on the utilization of ICT in various sectors. Building on government’s commitment, health sector decision-makers also should use the ICT more to allow the hard-to-reach population to reach the services quickly and improve supervision and monitoring. Worth mentioning is the fact that the health sector has already achieved much success in this regard; however, further work is recommended.

*(10) Improve Health Promotion and Disease Prevention.* In regard to the importance of SBCC in achieving UHC, a key informant from a multilateral organization remarked:

“If we are able to provide good health promotion, prevention, and we are able to bring a change on the behavior of the people that could have a significant impact later on cost of the services so that we avoid expensive interventions.”

*(11) Deciding on and Adhering to Quality Criteria.* Strict criteria for quality of care should be set, and a directive should be passed that providers would receive payment only if they comply with an agreed treatment protocol and quality criteria. Functioning referral mechanism should be ensured, along with a defined referral protocol. These require improved capacity of service providers, which can be attained through proper training. The training should not be limited to technical aspect of care but rather should include training on responsiveness or patient-centered care and quality of care. An advisor to an international NGO said

“A physician should be psychologically prepared in medical colleges, where they stay for five years. Our curriculum lacks issues like what should be the appropriate attitude in the profession they (students) are going, what should be their behavior. . . .There should be a review of the medical curriculum to orient them about the environment where they would be working. These should be included in medical education, or if not possible, then at least in the orientation trainings.”

*(12) Code of Conduct for Service Providers.* There should be code of conduct for service providers, like physicians and nurses. These need to be developed in consultation with relevant stakeholders, including professional bodies of the respective professional groups, i.e., Bangladesh Medical Association (BMA), Bangladesh Diploma Nurses Association (BDNA), etc.

*(13) Improve Efficiency.* To best utilize the existing resources, technical and allocative efficiency should be ensured. Decision-makers should push for improved taxmanagement; managers should decide on priory expenditures. Costing analysis of all services is needed, which demands developing a costing unit under HEU.

*(14) Special Attention to Hard-to-Reach Areas and Marginalized Populations.* Special attention should be paid to hard to reach areas and the marginalized population. A key informant from the local level family planning directorate of the government said

“Ifa mother has five children, she tries to help out the weakest one. Similarly, we should identify the areas that are weak socioeconomically; and focus more from the policy perspective.”

*(15) Decentralization.* A policy reform for decentralization is needed. A key informant from an international NGO said

“Wherever we engaged local government, we were met with success. It is true for service utilization, reaching the hard to reach, inclusion of poor people – in all of these areas local government was very instrumental.”

Suggestions to Address Demand-Side Barriers

*(16) Patient/Client Education.* We need to inform people about UHC in order to generate demand for it. We need to improve health literacy of general population, which is not possible only by the health sector. Health literacy should be enhanced through the general education as well. Common people should know what services are available.

*(17) Community Empowerment.* Communities should know what they are entitled to and how to get the responsible persons accountable for their work.

Suggestions to Address Crosscutting Barriers

*(18) Research.* In order to improve our knowledge and under standing on UHC, particularly in the context of Bangladesh, further research is necessary. The research should address the current pattern of health care expenditure, equity status of health care, different models of healthcare financing, various other issues related to different aspects of health systems, relevant to UHC. A health systems practitioner from Sylhet said

“We need to identify why physicians are not going to the place where they are posted. If you give a physician posting to Sulla (a difficult place), why they don’t stay there? We need to understand that first. I have been there, I know, if you are posted there, you will feel like, you are not even in this country.”

*(19) Advocacy.* Based on research, policy best-practices, and multisectoral experiences, advocacy for UHC should continue.

## 4 Discussion

This study found that almost all relevant policy documents of GoB explicitly acknowledged the need for UHC and proposed some directives. Many policy directives have already been translated into actions, by the central and local level public sector, NGOs, multilateral organizations and donors, academic and research organizations, and the media, in alignment with the ESP. However, there are barriers towards achieving UHC. Some barriers pertain to larger policy level, often beyond the health sector, while others pertain to the health sector’s implementation underperformances. The issue of demand-side barriers has also been raised by most of the key informants. Some empirical suggestions have also been made by the real practitioners, to tackle the barriers towards UHC.

Rockefeller Foundation’s Transforming Health Systems funding program in 2010 was one of the first initiatives to jumpstart UHC activities in Bangladesh. This multipronged funding approach involved building capacity, generating evidence, improving health information system, involving the media, improving medical education, and raising awareness, which eventually contributed to the overall policy preparedness and buy-in by the government, as well as the relevant civil society [32]. This early momentum was supplemented by the Prime Minister Sheikh Hasina’s commitment to achieve UHC by 2032, which she declared in the 64 United Nations General Assembly in May 2011 [33]. These, supposedly, culminated into the policy level commitment of Bangladesh for UHC, as reflected in our study as well.

Despite commitment by the policy makers as well as its reflections in policy documents such as the Health Care Financing Strategy 2012-2032 and Communication Strategy for UHC 2014-2016, many barriers exist. The issue of rigid colonial-era public financial management structure has been mentioned as an important barrier to UHC. This issue has been relatively less discussed in the existing health policy literature of Bangladesh, except for a recent commentary by a high level health bureaucrat and colleagues [34]. Further research, especially garnering the views from the Ministry of Finance in this regard would be useful. The issues of governance shortfall are also under-researched, especially in the context of their role in UHC. The role of budgetary deficiency, which is 4.3% of the total budget in 2015-2016, has also been pointed out in other studies 6 Ahmed, Evans, Standing, and Mahmud [35] argued in favor of a strong regulatory and mediatory mechanism in Bangladesh, which is currently experiencing a rapid growth in private sector healthcare.

Implementation barriers, in relation to UHC, are relatively better represented in the health system literature from Bangladesh. Different aspects of health workforce crisis (e.g., absolute shortage, under performance, skill-mix imbalance, difficulty in rural retention, lack of responsiveness) have been widely documented by Ahmed, Hossain, Chowdhury, and Bhuiya [36], Rawal, Joarder, and Mahmud [37], and Joarder, George, Sarker, Ahmed, and Peters [38] among others. The World Bank commissioned a full-fledged study examining the human resources for health (HRH) issues related to UHC in Bangladesh, in which they identified the challenges, and proposed specific policy options 29Lapse in monitoring and supervision, especially that in case of HRH, has been pointed out in other studies too [3, 29]. Mehl and Labrique [39] suggested prioritizing integrated health strategies to improve the monitoring and evaluation, drawing upon examples from India and Bangladesh. The deficiency of quality of care in Bangladesh and the need for a guideline and commensurate training of the service providers have also been argued in many studies [40–44]. The need for flexible budgeting and local level planning too have been advocated in multiple policy documents and studies [3, 28, 34].

Studies on demand-side issues pertaining to UHC are available, but not abundant, like the ones pertaining to implementation issues mentioned above. Ahmed et al. [45] explored the willingness to pay for community based health insurance among informal workers in urban Bangladesh. 86.7% of the informal workers, such as the rickshaw pullers, restaurant workers, and shopkeepers expressed their willingness for such insurance. Joarder, Uddin, and Islam [46], in a mixed-methods study from rural Bangladesh, argued community empowerment to be a cornerstone of UHC and delineated the status of community empowerment in terms of access to information, inclusion and participation, ability to hold decision makers accountable, and local organizational capacity. They found 90% of the respondent had access to some sources of health information, but the other aspects of community empowerment were almost nonexistent. Trust in the health system has been argued to be an important factor to establish a prepayment based health-financing mechanism [47] and other aspects of service provision [48–51], which are essential for UHC. There is no research on historical mistrust and its relation to religiocultural disinclination of Bangladeshis towards prepayment-based health financing. Further research is needed to understand this phenomenon better.

In order to overcome these barriers, several policies and strategies have also been proposed. These include, but are not limited to, redesigning the public financial management structure, introducing health-financing reform, improving regulatory and mediatory mechanisms, embracing inter sectoral collaboration, and garnering political commitment for UHC. In order to address the implementation barriers, key informants proposed strengthening the overall health systems, improving the health service management, improving monitoring and supervision, adhering to service quality criteria, specifying code of conduct for service providers, etc. Patient education and community empowerment have been proposed to address the demand-side barriers. A need for research and advocacy has also been pronounced, to address the crosscutting barrier, i.e., lack of common understanding on UHC.

Despite sincere efforts from the researchers, the study had some limitations, such that the study could not achieve data saturation. We had to limit the number of respondents due to time and resource constraints. Due to the same reason, further variety in selection of stakeholder respondents could not be entertained. Of particular importance is the view of the Ministry of Finance (MoF), which is one of the most important stakeholders and decision makers in matters of financing for UHC. It is recommended that further research and advocacy involving the MoF stakeholders are undertaken. A detailed prospective policy analysis in favor of UHC is also highly recommended.

## 5 Conclusions

The policy environment in Bangladesh is primed for an all out progress towards UHC, especially owing to the commitment from the highest political level within the country. However, outdated public financial management structure, coupled with demand-side barriers (e.g., sociocultural disinclination, lack of citizen empowerment and demand) pose a serious threat to the timely realization of UHC goals and aspirations. Therefore, customized and context-specific policy adjustments need to be incorporated for progress towards UHC and subsequently achieving the pertinent SDGs. Having identified the challenges and potential solutions, it is believed that Bangladesh will accomplish its mission successfully towards equity and UHC.

## Data Availability

The qualitative data used to support the findings of this study are included within the article and the supplementary information file. The transcripts are available from the corresponding author upon request.

## Disclosure

The funders did not have any role in design of the study and collection, analysis, and interpretation of data and in writing the manuscript.

## Conflicts of Interest

The authors declare that there are no conflicts of interest regarding the publication of this paper.
