# Supplementary material for: Universal Health Coverage in Bangladesh: Activities, Challenges, and Suggestions
Source: Psyche (Camb Mass). 2019 Mar 3;2019:4954095. doi: 10.1155/2019/4954095 (PMC7691757; doi:10.1155/2019/4954095)
Supplement: Supplementary file 1 [file APH-2019-4954095-s001.docx]

**Additional information file 1:** **Summary of policy documents related to Universal Health Coverage in Bangladesh**

| **Name of the document, year of publication, and publishing authority** | **Main findings** |
| --- | --- |
| **Overarching GoB documents, not specific to health sector** | |
| Seventh Five-Year Plan Fiscal Year 2016-2020: Accelerating Growth, Empowering Citizens. December 2015. General Economic Division, Planning Commission. | Dedicated Chapter 10 for health sector and proposed the Health, Nutrition, and Population Development Strategy. Placed a special emphasis on protecting the interest of the poor in service delivery. Committed to ensure that the poor and the marginalized people are able to access and utilize health services. Health financing reform has been stated as one of the commitments of serving the poor population. Acknowledged the existing deficiency in per capita health expenditure, deficiency in the share of the national budget for health, high OOP, and lack of quality of care. In order to tackle these issues, it referred to the Health Care Financing Strategy (2012-2032) for solutions. |
| National Social Security Strategy (NSSS) of Bangladesh. July 2015. General Economic Division, Planning Commission. | Acknowledged the importance of health financing reform in curbing health shocks. Proposed some reforms and action plans, and listed relevant ministries to collaborate with. Expressed the commitment of the GoB to introduce health insurance, which has also been acknowledged as an example of a health sector program that closely complements social security initiatives. |
| National Health Protection Act (draft). 2014. National Health Protection Authority, Ministry of Health, and Family Welfare. | Acknowledged the importance of sustainable health financing option that entails to strengthen financial risk protection, and extend health services and population coverage, with an aim to achieve UHC. This Act has been envisioned by the MoHFW to serve as the legal basis for new health financing mechanism, and provide a legal framework to GoB’s UHC targets. |
| Shasthyo Shandhan (Search for Health): A Guideline on Universal Health Coverage for The Mass Media. 2016. Press Institute of Bangladesh. | Developed with financial assistance from the Rockefeller Foundation, in order to generate awareness among journalists on UHC. Easy-to-comprehend document written in Bengali, covering the basic concepts of health, health governance structure of Bangladesh, health care structure, challenges in health sector, and health financing issues. Provides information on the concept of UHC, experiences of other countries (e.g., Thailand, India, Brazil, and China) with UHC, feasible options for Bangladesh, etc. |
| **Overarching GoB documents specific to health sector, but not specific to health financing** | |
| National Health Policy 2011. January 2012. Ministry of Health and Family Welfare. | Admitted the insufficient budgetary allocation in health sector and recommended increasing it. In Strategic Principle part, proposed ensuring equitable care for the disadvantaged, poor, marginalized, elderly, and disabled population. In ‘Strategies’ part, proposed health insurance for formal sector employees, which would later be extended gradually to other groups. For ultra-poor and disadvantaged populations, proposed free health services. |
| Health Population and Nutrition Sector Strategic Plan (HPNSSP) 2011 – 2016. September 2010. Planning Wing, Ministry of Health and Family Welfare. | Dedicated a chapter on ‘health sector financing’, where it proposed a health-financing framework, advocated demand-side financing, and proposed a resource allocation formula. Stated specific purpose in relation to health financing to make sufficient funding available to ensure that all individuals have access to effective public health and personal health care. Proposed some priority activities, which included identify financing constraints; assess the strengths and weakness of the existing financing mechanism; assess suitability of different financing schemes; review examples from other countries, etc. |
| Health Nutrition and Population Strategic Investment Plan (HNPSIP) 2016-2021. April 2016. Planning Wing, Ministry of Health and Family Welfare. | Identified ‘essential health service package’ as the first milestone on the road to UHC. Clearly outlined three guiding principles for attaining UHC: quality, equity, and efficiency across health services. Identified 10 driving forces for HNPSIP, the final one of which suggested greater investment in health, ensuring a focus on managing demand, increasing efficiency, and developing the evidence base for future health funding. Proposed eight strategic objectives; the third of which was “To provide sustainable financing for equitable access to health care for the population and accelerated progress towards universal health coverage.” This strategic objective, envisioned to achieve three outputs: 1. Increase overall financial resources in the health sector; 2. Achieve equitable access to services and financial protection, especially for people in the bottom 40 percent; and 3. Enhance efficiency in financial resource allocation and use. Going one step further, outlined priority activities in order to achieve the proposed outputs. |
| Monitoring and Evaluation Strategy and Action Plan: Health Population and Nutrition Sector Development Program (HPNSDP). December 2014. Program Management and Monitoring Unit, Planning Wing, Ministry of Health and Family Welfare. | Did not explicitly discuss UHC; but it proposed monitoring and evaluation plans for financial progress for different operational plans. We felt that the monitoring and evaluation plans should have included components of equity, so that, programs can be evaluated for equity gains (or losses) as well. |
| **GoB documents specifically related to health financing** | |
| Health Care Financing Strategy 2012-2032: Expanding Social Protection for Health towards Universal Coverage. September 2012. Health Economics Unit, Ministry of Health and Family Welfare. | The most important policy documents for UHC. Aligned with the vision of HPNSDP 2011-2016, the UHC, and the National Health Policy 2011; and acknowledged the importance of bringing more funds to the health sector and pooling the resources effectively. Summarized challenges of health financing in Bangladesh as: 1. Inadequate health financing; 2. Inequity in health financing and utilization; and 3. Inefficient use of existing resources. Designed to address the health financing issues for the next 20 years, proposed ways to combine funds from tax-based budgets with proposed social health protection schemes (including for the poor and the formal sector), existing community based and other pre-payment schemes and donor funding to ensure financial protection against health expenditures for all segments of the population, starting with the poorest. Recognized the importance of and proposed collaboration with the for-profit and not-for-profit private sector, development partners, and the community people; to resolve the health financing challenges. Proposed a gradual process to achieve universal coverage, starting from the poor and the formal sector (public, for-profit private, and not-for-profit private), progressively to remaining segments of the population by 2032. Proposed three strategic objectives: 1. Generate more resources for effective health services; 2. Improve equity and increase healthcare access, especially for the poor and the vulnerable; and 3. Enhance efficiency in resource allocation and utilization. Proposed three strategic interventions and supportive actions: 1. Design and implement a Social Health Protection Scheme; 2. Strengthen financing and provision of public healthcare services; and 3. Strengthen national capacity. |
| Framework for Monitoring Progress Towards Universal Health Coverage in Bangladesh. 2014. Health Economics Unit, Ministry of Health and Family Welfare. | Developed a set of indicators, with technical support of the WHO Bangladesh country office, to monitor the progress towards UHC. There were 43 indicators, covering four areas: 1. Access to health services, 2. Protection against, financial risk, 3. Population coverage, and 4. Quality of services. |
| Operational Mechanism for Social Health Insurance in Poverty Prone Sub-districts of Bangladesh: Developing Tools and Guidelines. March, 2005. Health Economics Unit, Ministry of Health and Family Welfare. | Described the mechanisms of the social health insurance that GoB is currently piloting in two sub-districts of Bangladesh. Discussed constraints and remedies of social health insurance in Bangladesh, basic principles and practices of commercial insurance in the context of Bangladesh, and the problems involved in community based health insurance. |
| Bangladesh National Health Accounts (BNHA)-V 1997 – 2015. September 2015. Health Economics Unit, Ministry of Health and Family Welfare. | Important policy tool to decide the distribution of societal resources for healthcare as it provided information on different aspects of total health expenditure, and monitor the flow of funds at different levels. Showed that, 23% of total health expenditure (THE) is shared from public, 67% from OOP, and the rest 10% from other sources. Provides a functional classification of THE; where the highest percentage was spent on medical goods (46.6%), followed by services of curative care (25.3%), preventive care (10.9%), ancillary services (5.5%). Only 2% goes for education, research, and training of health personnel. |
| **GoB documents not directly related to, but with implications for UHC** | |
| Strategic Planning on Quality of Care for Health Service Delivery in Bangladesh. January 2015. Quality Improvement Secretariat, Ministry of Health and Family Welfare. | Recognized that the issue of quality of care (QoC) has become more important in the context of UHC, as it requires optimizing the resource use and expanding coverage with QoC. Set the basis for a focused and coordinated framework or implementing quality improvement activities. Proposed strategic objectives: 1. Introduce consumer and patient-centered services; 2. Improve patient safety; 3. Improve clinical practice; 4. Improve leadership management systems; 5. Improve public health and preventive services; 6. Ensure all necessary inputs for quality improvement; 7. Ensure all necessary support services; and 8. Develop effective outcome measurement system for quality improvement. |
| Strengthening Stewardship Functions of the Regulatory Bodies under MoHFW. August 2014. Ministry of Health and Family Welfare. | Reviewed the stewardship and governance functions of the regulatory bodies under the MoHFW. Suggested recommendations for these bodies in order to increase effectiveness of the regulatory functions including updating the mandate and structure of the regulatory bodies. Proposed feasible action plans in implementing the recommendations with possible sources of funding. Their review included all the statutory and professional regulatory bodies including national regulatory bodies such as Bangladesh Medical and Dental Council, Bangladesh Nursing Council, Pharmacy Council of Bangladesh, State Medical Faculty, etc.; and various MoHFW directorates such as Directorate General of Health Services, Directorate General of Family Planning, and Directorate General Drug Administration. |
| Bangladesh Essential Service Package, August 2016. Ministry of Health and Family Welfare. | Described itself as a powerful tool for UHC, as it could help selecting the services that should be made available to the whole population as a guaranteed minimum, thus enhancing equity. Stated that Essential Service Package (ESP) provision should prioritize hard to reach and vulnerable population, and be sustainable in the long run. The three purposes of ESP, as outlined by the MoHFW, were claimed to be aligned with the realization of UHC. These three purposes were: 1. Associated with UHC initiatives, the ESP represents the GoB commitment to ensure the right to health and that the whole population has access to most essential health services; 2. The ESP will become the basis to define the set of standards by type of health facility; and 3. The package, common to the whole territory, is to be used for resource allocation in a way that promotes equity and increases efficiency. |
| Bangladesh Health Workforce Strategy. 2015. Human Resource Management Unit. Ministry of Health and Family Welfare. | Summarized the issues and challenges related to human resources for health (HRH) in Bangladesh under five thematic areas with one strategic objective for each: 1. Health workforce planning: Make available, competent and adequate number of workforce as per health systems need; 2. Health workforce capacity development: Produce, develop, and sustain quality health workforce at all levels; 3. Health workforce deployment, retention, and professional engagement: Recruit, deploy, and retain health workforce equitably; 4. Management of high performance standards: Promote and maintain high standards in health workforce performance; and 5. Health workforce information system: Promote evidence-based health workforce decision-making in improving health outcomes. The strategy was underpinned by four guiding principles: 1. Gender balance, 2. Motivation, 3. Partnership, and 4. Transparency and accountability. |
| Health Bulletin. 2016. Management Information System, Directorate General of Health Services. Ministry of Health and Family Welfare. | Flagship publication of Directorate General of Health Services, highlighting the overall health scenario in Bangladesh. Useful source of information in regards to UHC activities, status, and progress in Bangladesh. |
| **Documents related to UHC, not published by GoB** | |
| Bangladesh Health Watch Report 2011: Moving Towards Universal Health Coverage. 2012. James P Grant School of Public Health, BRAC University. | Multi-organization civil society network to hold the state and non-state sectors accountable for their performance in delivering quality health care to the citizens. Commissioned several studies to investigate Bangladesh's preparedness to achieve UHC within the foreseeable future, and identify opportunities as well as challenges, which need to be overcome. Looked at Bangladesh's experiences with public sector in-patient health care; demand side financing; health care financing by NGOs; and for profit private health insurance. Messages emerging from these studies included: 1. The need to establish a shared understanding of universal coverage; 2. Two dimensions are important: a comprehensive set of quality services according to need and financial protection in accessing care ensuring that individuals are not economically compromised in paying for care. Drew seven overarching recommendations: 1. Establishing a shared understanding of UHC; 2. Making a strong case for UHC; 3. Moving to pre-payment financing is imperative; 4. Educating and empowering beneficiaries; 5. Accelerating provision of more equitable and efficient health services; 6. Transforming information and evidentiary systems; and 7. Acquiring core competencies for UHC. |
| The Path to Universal Health Care in Bangladesh: Bridging the Gap of Human Resources for Health. 2015. World Bank. | Identified few challenges in terms of HRH in Bangladesh, HRH policy challenges, as well as policy options for UHC in relation to HRH. HRH challenges were identified to be: 1. Shortages, 2. Production shortfalls, 3. High vacancy rates and slow recruitment, 4. Skill-mix imbalances, 5. Urban and gender biases, 6. Quality of healthcare provision and productivity of healthcare providers, and 7. Work environment. HRH policy challenges included: 1. A complex array of national policies; 2. A highly centralized and cumbersome bureaucratic system with weak response capacity; 3. A range of powerful stakeholders, some with competing interests; and 4. Weak regulatory and enforcement capacity, contributing to high rates of absenteeism and many unqualified health workers. HRH policy options for UHC included: 1. Address HRH shortages; 2. Improve the skill-mix; 3. Address geographic imbalances; 4. Retain health workers; 5. Adopt strategic payment and purchaser mechanisms; 6. Establish a central human resources information system; 7. Target HRH interventions to improve maternal and newborn health. Several strategies have also been proposed under each policy options. |
| Bangladesh Health System Review. 2015. World Health Organization. | Provided a detailed description of health system and of policy initiatives of Bangladesh. Examined approaches to the organization, financing and delivery of health services and the role of the main actors in health systems; described the institutional framework, process, content, and implementation of health and health-care policies; and highlighted challenges and areas that required more in-depth analysis. Identifies some critical challenges for the health system: 1. Lack of coordination across two different ministries for implementing primary healthcare service delivery in rural and urban areas; 2. Critical shortage of trained health providers with appropriate skill-mix in the public sector and widespread increase in unregulated informal providers for an alternative source of care; 3. Low annual allocation to health in the government budget and high out-of-pocket payments by households; and 4. Inequitable access to health services between urban and rural areas including variable health financing mechanisms, which have slowed achieving UHC. |
| Health Equity and Financial Protection Datasheet: Bangladesh. 2012. World Bank. | Provided a picture of equity and financial protection in the health sector of Bangladesh. Covered topics included: 1. Inequalities in health outcomes, health behavior, and health care utilization; 2. Benefit incidence analysis; 3. Financial protection; and 4. The progressivity of health care financing. |
| Bangladesh Demographic and Health Survey 2014: Key Indicators. 2015. National Institute of Population Research and Training (NIPORT), Mitra and Associates, ICF International. | Nationwide sample survey, the seventh of its kind in Bangladesh, of ever-married women of reproductive age designed to provide information on: 1. Fertility and childhood mortality levels; 2. Fertility preferences; 3. Use of family planning methods; 4. Maternal, newborn, and child health including breastfeeding practices; 5. Nutritional status of under-5 children; 6. Knowledge and attitudes toward HIV/AIDS and other sexually transmitted infections (STI); and 7. Community-level data on accessibility and availability of health and family planning services. This up-to-date information was intended to assist policymakers and program managers in evaluating HPNSDP and in designing programs and strategies for improving health and family planning services in the country. |
| **Articles published in Lancet Series­– ‘Bangladesh: Innovation for Universal Health Coverage’** | |
| The Bangladesh paradox: exceptional health achievement despite economic poverty | Argued that the exceptional performance of Bangladesh in health sector might be attributed to a pluralistic health system that had many stakeholders pursuing women centered, gender-equity-oriented, highly focused health programs in family planning, immunization, oral rehydration therapy, maternal and child health, tuberculosis, vitamin A supplementation, and other activities, through the work of widely deployed community health workers reaching all households. Government and NGOs had pioneered many innovations that had been scaled up nationally. Bangladesh offers lessons such as how gender equity can improve health outcomes, how health innovations can be scaled up, and how direct health interventions can partly overcome socioeconomic constraints. |
| Harnessing pluralism for better health in Bangladesh | Suggested key areas for managing pluralism: 1. Participatory governance; 2. Accountability and regulation; 3. Information systems; and 4. Capacity development. |
| Community-based approaches and partnerships: innovations in health-service delivery in Bangladesh | Identified three distinctive features that enabled Bangladesh to improve health-service coverage and health outcomes: 1. Experimentation with, and widespread application of, large-scale community-based approaches, especially investment in community health workers using a doorstep delivery approach; 2. Experimentation with informal and contractual partnership arrangements that capitalize on the ability of NGOs to generate community trust, reach the most deprived populations, and address service gaps; and 3. Rapid adoption of context-specific innovative technologies and policies that identify country-specific systems and mechanisms. |
| Explaining equity gains in child survival in Bangladesh: scale, speed, and selectivity in health and development | Discovered that there remain significant residual inequities in survival of girls and lower wealth quintiles as well as a host of new health and development challenges such as urbanization, chronic disease, and climate change. According to the authors, further progress requires stronger governance and longer-term systems thinking that addresses health workforce shortages, shortfalls in effective coverage of services, and enhanced engagement of partners within and beyond the health system. |
| Reducing the health effect of natural hazards in Bangladesh | Was on impact of natural disaster in Bangladesh, and argued that urbanization and climate change created new challenges, and earthquakes remains an unaddressed threat. |
| Innovation for universal health coverage in Bangladesh: a call to action | Proposes a pragmatic reform agenda for achieving UHC in Bangladesh: 1. Development of a long-term national human resources policy and action plan; 2 Establishment of a national insurance system; 3. Building of an interoperable electronic health information system; 4. Investment to strengthen the capacity of the MoHFW; and 5. Creation of a supra-ministerial council on health. Short (1-5 years) and medium (5-15 years) term actions commensurate with each agenda have also been proposed. |

**Additional information file 2: Key Informant Interview Tools**

**Universal Health Coverage: Status, Challenges, Recommendations and Commitments**

**Tool for Local Level Respondents**

1. Three dimensions of Universal Health Coverage (UHC): Population coverage, service coverage, cost coverage. Could you tell us about activities that are being done in your area in line with these three dimensions?

- Probe: Anything on the coverage of the women, children, and adolescents?
- Probe: What about quality of care?

2. Please tell us about your work, which might be contributing to the UHC agenda.

- Probe: Anything that may contribute to quality aspect?
- Probe: Anything that may contribute to improved cost coverage?

3. Do you face or feel any barriers in your work (pertaining to the works you mentioned to be contributing to UHC)?

4. What policy level changes do you want to see in the country’s health sector in order to move towards UHC?

- Probe: Any policy change to increase the coverage for women, children, and adolescents?
- Probe: Any policy change to facilitate people accessing services with less financial barriers?

5. What would be your commitment, in this area (Sylhet district or the upazila under the district) in particular, to move towards UHC?

- In terms of population coverage (equity)
- In terms of service coverage (including quality of care)
- In terms of cost coverage (decreasing direct payment or OOP)

6. Anything else that you want to add?

**Universal Health Coverage: Status, Challenges, Recommendations and Commitments**

**Tool for Central Level Respondents**

1. Three dimensions of Universal Health Coverage (UHC): Population coverage, service coverage, cost coverage. What is your organization’s position and activities in addressing these three dimensions?

- Probe: Anything on the coverage of the women, children, and adolescents?
- Probe: What about quality of care?

2. What are the barriers towards achieving UHC?

- Probe: Reasons for exclusion of women, children, and adolescents
- Probe: Why quality of services are compromised for certain groups

3. What policy level changes do you want to see in the country’s health sector in order to move towards UHC?

- Probe: Any policy change to increase the coverage for women, children, and adolescents?

4. What would be, as a leader of your organization, your role and commitment, say in next one year, to move towards UHC?

- In terms of population coverage (equity)
- In terms of service coverage (including quality of care)
- In terms of cost coverage (decreasing direct payment or OOP)

5. Anything else that you want to add?
